# Novel Activity of a Synthetic Decapeptide Against *Toxoplasma gondii* Tachyzoites

**DOI:** 10.3389/fmicb.2018.00753

**Published:** 2018-04-20

**Authors:** Laura Giovati, Claudia Santinoli, Carlo Mangia, Alice Vismarra, Silvana Belletti, Tiziana D’Adda, Claudia Fumarola, Tecla Ciociola, Cristina Bacci, Walter Magliani, Luciano Polonelli, Stefania Conti, Laura H. Kramer

**Affiliations:** ^1^Laboratory of Microbiology and Virology, Department of Medicine and Surgery, University of Parma, Parma, Italy; ^2^Department of Veterinary Science, University of Parma, Parma, Italy; ^3^Laboratory of Histology, Department of Medicine and Surgery, University of Parma, Parma, Italy; ^4^Laboratory of Pathological Anatomy, Department of Medicine and Surgery, University of Parma, Parma, Italy; ^5^Laboratory of Experimental Oncology, Department of Medicine and Surgery, University of Parma, Parma, Italy

**Keywords:** anti-*Toxoplasma* agents, killer peptide KP, apoptosis, TUNEL, mitochondrial potential, transmission electron microscopy

## Abstract

The killer peptide KP is a synthetic decapeptide derived from the sequence of the variable region of a recombinant yeast killer toxin-like microbicidal single-chain antibody. KP proved to exert significant activities against diverse microbial and viral pathogens through different mechanisms of action, but little is known of its effect on apicomplexan protozoa. The aim of the present study was to evaluate the *in vitro* activity of KP against *Toxoplasma gondii*, a globally widespread protozoan parasite of great medical interest. The effect of KP treatment and its potential mechanism of action on *T. gondii* were evaluated by various methods, including light microscopy, quantitative PCR, flow cytometry, confocal microscopy, and transmission electron microscopy. In the presence of KP, the number of *T. gondii* tachyzoites able to invade Vero cells and the parasite intracellular proliferation were significantly reduced. Morphological observation and analysis of apoptotic markers suggested that KP is able to trigger an apoptosis-like cell death in *T. gondii*. Overall, our results indicate that KP could be a promising candidate for the development of new anti-*Toxoplasma* drugs with a novel mechanism of action.

## Introduction

Toxoplasmosis, a globally widespread zoonotic disease that affects a variety of mammals, including humans, is caused by *Toxoplasma gondii*, an obligate intracellular parasite estimated to infect at least one-third of the world population (Tenter et al., 2000; Flegr et al., 2014; Hill and Dubey, 2016). Infection in immunocompetent subjects, acquired through ingestion of meat containing tissue cysts or food and water contaminated with oocysts released by infected felines, is generally subclinical (Tenter et al., 2000; Pereira et al., 2010; Hill and Dubey, 2016). Host immune response does not eradicate the parasite but promotes its conversion from a rapidly replicating tachyzoite form to a quiescent encysted bradyzoite stage that persists lifelong. In immunocompromised persons, especially those with AIDS, a reactivation of latent infection can occur, leading to significant morbidity and mortality (Blader et al., 2015; Hill and Dubey, 2016). Mother-to-child transmission during primary infection of pregnant women can lead to congenital toxoplasmosis, with clinical manifestations of varying severity (McAuley, 2014; Blader et al., 2015).

A combination of pyrimethamine and sulfadiazine represents the gold-standard chemotherapy for toxoplasmosis. These compounds are highly effective against acute infection, but have many side effects; furthermore, current chemotherapy is not completely effective in eradicating encysted bradyzoites and in treating congenital toxoplasmosis (Montazeri et al., 2017). Several candidates have been proposed for the development of immunoprophylactic strategies against toxoplasmosis, but research is still ongoing (Zhang et al., 2015; Hill and Dubey, 2016). Thus, alternative therapeutic compounds, with novel mechanisms of action against *T. gondii* and non-toxic, are needed.

Diverse natural and synthetic antimicrobial peptides, whose mechanism of action involve damage to cellular membranes and killing by osmotic lysis, showed good antiprotozoal activity and low toxicity to mammalian cells (Mor, 2009; Torrent et al., 2012). Other antimicrobial peptides interact with intracellular targets, inducing parasite death in a manner similar to that observed during autophagic or apoptotic death in mammalian cells (Bera et al., 2003; Delgado et al., 2008; Kulkarni et al., 2009; Rathore et al., 2011). Most of the described antiparasitic peptides acted on *Plasmodium* and *Leishmania* species. Studies on the activity of defensin-like peptides against *T. gondii* demonstrated a mechanism of killing through membrane pore formation (Tanaka et al., 2010, 2012).

The killer decapeptide KP derives from the sequence of the variable region of a single-chain recombinant anti-idiotypic antibody representing the internal image of a yeast killer toxin characterized by the wide spectrum of antimicrobial activity (Polonelli et al., 2003). A number of previous studies proved the efficacy of KP against different pathogens, including extracellular protozoa, i.e., trophozoites of *Acanthamoeba castellanii* and *Leishmania* spp. promastigotes (Magliani et al., 2011; Ciociola et al., 2015).

The aim of the present study was to evaluate the effect of KP on extracellular tachyzoites of *T. gondii* in an *in vitro* model and to explore its potential mechanism of action.

## Materials and Methods

### Peptides

The decapeptide KP (AKVTMTCSAS) (Polonelli et al., 2003) was synthesized by NeoMPS (PolyPeptide Group, Strasbourg, France). The scrambled synthetic peptide SP (MSTAVSKCAT), containing the same amino acids in a different sequence, was used as negative control. Peptide purity (HPLC analysis) was 97.4% for KP and 95.8% for SP. KP and SP were solubilized in DMSO (20 mg/mL) and diluted prior to use. In all experiments, controls (without peptides) contained DMSO at the proper concentration.

### Cytotoxicity Assay

Cytotoxicity of KP against Vero cells (ECACC 84113001) was determined by 3-(4,5-dimethyl-2-thiazolyl)-2,5-diphenyl-2H-tetrazolium bromide (MTT) assay. Vero cells cultured in RPMI 1640 containing 2 mM L-glutamine, 100 U/mL penicillin, and 0.1 mg/mL streptomycin (complete medium), added with 10% heat-inactivated FBS, were seeded in 96-well plates (1 × 10^5^ cells/mL, 100 μL/well) and incubated for 24 h at 37°C in 5% CO_2_ atmosphere. Cells were then incubated for 24 h in medium containing 2% FBS in the absence (control) or presence of KP (final concentrations 50, 100, and 200 μg/mL). MTT (5 mg/mL, 10 μL/well) was then added in 100 μL serum-free medium for 2 h at 37°C. Formazan crystals formed by viable cells by reduction of MTT were solubilized in 100 μL acidified isopropanol and absorbance was measured at 540 nm. Each assay was performed in triplicate. Results, from two independent experiments, are expressed as percentage of viable cells in comparison to control.

### Propagation of *T. gondii* Tachyzoites

Tachyzoites of RH strain, Type I, maintained in Vero cells cultured as previously described into 75 cm^2^ flasks, were harvested directly before use.

### Evaluation of KP Effect on the Invasion and Intracellular Proliferation of *T. gondii* in Vero Cells

Vero cells, cultured as previously described on eight-well chamber slides (1.5 × 10^4^ cells/well, 200 μL) for 24 h, were infected with five tachyzoites/cell. At the same time, KP was added (200, 100, 50, and 25 μg/mL). Cells infected without KP or added with SP (200 μg/mL) served as control. After 3 h the medium was replaced with fresh RPMI (2% FBS) without peptides. After 72 h, cells were washed in PBS to remove non-adherent parasites, fixed in 10% buffered formalin for 24 h, and stained with a modified Giemsa (Diff-Quick Stain, Bio-Optica) prior to microscopic observation. Infection index and parasite intracellular proliferation (number of infected cells and total number of parasites/200 examined cells, respectively) were assessed. Three slides for each condition were evaluated by two independent observers. Results are expressed as mean values ± SD and as percentages of inhibition in comparison to control (medium alone).

### Evaluation of KP Effect on *T. gondii* Replication by Quantitative Real-Time PCR

Vero cells were cultured on two 12-well plates (1 × 10^5^ cells/well, 200 μL), infected with tachyzoites, and treated with KP or SP as previously described. After 3 h the medium was replaced with fresh RPMI (2% FBS) without peptides. At 3, 24, 48, and 72 h post-infection, tachyzoites were collected, the medium was removed, each well washed twice with Hank’s Solution, and the fluids harvested in a 15 mL tube. Vero cells were detached with Trypsin/EDTA and centrifuged together with the tachyzoites suspensions at 1800 *g* for 10 min. DNA was extracted from pellets suspended in PBS using the DNeasy Blood & Tissue Kit (Qiagen), according to manufacturer’s instructions, and quantified spectrophotometrically. The amount of parasite DNA in each sample was determined by a quantitative real-time PCR, using the SsoAdvanced SYBR Green Supermix (Bio-Rad) with primers (forward 5′-CACAGAAGGGACAGAAGT-3′, reverse 5′-TCGCCTTCATCTACAGTC-3′) targeting a 94-bp internal fragment in the 529-bp repeat element (Accession No. AF146527) of the parasite genome (Edvinsson et al., 2006). The assays were run in a CFX96 real-time machine (Bio-Rad) with a denaturation step (95°C for 5 min) and 45 repeated cycles (95°C–15 s; 58.5°C–30 s). The copy number of the sequences was determined using a standard curve made by subsequent 1:10 dilutions of a standard sample (detected range: 1.6 ng–1.6 pg). DNA extracted after 3 h of incubation was considered as the time zero (*T_0_*) condition. In all experiments, parasite quantification is expressed as DNA *vs.T_0_*. Three assays were run for each condition in two independent experiments.

### Evaluation of KP Effect on Extracellular *T. gondii* Tachyzoites

Extracellular tachyzoites (4 × 10^7^ cells/mL) were incubated for 3, 24, and 48 h in RPMI (2% FBS), in presence or absence of KP (50 or 100 μg/mL).

### Phosphatidylserine Exposure

Phosphatidylserine (PS) exposure was investigated with the Muse Cell Analyzer using the Muse Annexin-V & Dead Cell Assay kit (Merck Millipore), which utilizes Annexin-V to detect PS on the external membrane of apoptotic cells and 7-amino-actinomycin D (7-AAD) as a dead cell marker. KP-treated and control tachyzoites were handled according to manufacturer’s instructions. Percentages of cells in early (Annexin-V^+^/7-AAD^-^) and late (Annexin-V^+^/7-AAD^+^) apoptosis were determined. At least three independent experiments were performed.

### *In Situ* DNA Fragmentation Assay

DNA strand breaks in *T. gondii* tachyzoites were detected with a TUNEL (Terminal dUTP Nick End-Labeling) assay (Elmore, 2007). KP-treated and control tachyzoites, fixed in 2% paraformaldehyde for 1 h at 20°C, were suspended, after centrifugation, in ice cold permeabilization solution (0.1% Triton X-100 in 0.1% sodium citrate) for 2 min, then washed in PBS and incubated in TUNEL reaction mixture (Roche Diagnostics) for 1 h at 37°C in humidified atmosphere in the dark. Incorporation of labeled dUTP at DNA damaged sites was detected and quantified by a Beckman Coulter cytometer. A total of 10,000 cells were recorded from each sample. Two independent experiments, each including proper controls, were performed.

### Evaluation of Mitochondrial Membrane Potential by Confocal Laser Scanning Microscopy (CLSM)

The use of tetramethylrhodamine methyl ester (TMRM), a cationic probe that accumulates specifically in active mitochondria of viable cells in proportion to the magnitude of mitochondrial membrane potential (ΔΨm), was initially validated in *T. gondii*. Specific mitochondrial labeling and induction of detectable changes in ΔΨm with the mitochondrial uncoupler carbonyl cyanide-4-(trifluoromethoxy)phenylhydrazone (FCCP) were confirmed by confocal laser scanning microscopy (CLSM). Then, KP-treated tachyzoites were evaluated for changes in their ΔΨm after TMRM loading, either in end-point or in time-lapse CLSM studies through a LSM 510 Meta scan head integrated with an Axiovert 200M inverted microscope (Carl Zeiss) using a 63 × /1.4 oil objective (Ciociola et al., 2016). TMRM was excited with 543-nm He-Ne laser line. Transmitted light images were simultaneously recorded, to check tachyzoites morphology before and after KP treatment. For confocal observation in the end-point assays, after 18 h of incubation KP-treated and control tachyzoites (1 × 10^8^/mL) were suspended in an equal volume of melted 3% agarose containing 400 nM TMRM (final concentration). The suspension was solidified on a special chamber for confocal microscopy incubated for 40 min at 37°C in the dark. The chamber was lodged in a commercially available incubation system (Kit Cell Observer, Carl Zeiss) allowing the control of temperature and CO_2_ during image acquisition. Acquired images were processed for the semiquantitative analysis of ΔΨm variation, using the software developed by the microscope manufacturer. For each treatment condition, the average fluorescence intensity (FI) of sections acquired from six randomly selected fields was calculated. Data are presented as percent FI reduction in comparison to untreated control. For the time-lapse assay, 0.5 × 10^8^ tachyzoites/mL, embedded in 3% agarose containing 400 nM TMRM, were incubated on the confocal microscopy chamber at 37°C in the dark. Sequential images of a selected field were acquired before and at different times up to 3 h after KP addition. Tachyzoites in medium alone were assayed as a control.

### Transmission Electron Microscopy (TEM) Studies

Tachyzoites (1 × 10^8^ tachyzoites/mL) were incubated for 3 h in RPMI (2% FBS), in presence or absence of KP (50 or 100 μg/mL), then pre-fixed for 30 min with Karnovsky’s fixative and centrifuged at 1800 *g* for 10 min. Pellets were transferred into wells cut in solidified 3% agarose and covered with warm (56°C) agarose. After gelation, samples were fixed with Karnovsky’s fixative for 3 h at room temperature, then left overnight at 4°C. Post-fixation was performed with 1% osmium tetroxide for 45 min, followed by dehydration in acetone gradient (25–100%). Samples were infiltrated with multiple changes of Durcupan Araldite ACM1 epoxy resin, left overnight in ACM1, then embedded in Durcupan Araldite ACM2 resin, hardened by incubation for 72 h at 58°C. Semi-thin sections (0.75 μm) were stained with methylene blue and safranin and observed to assess the presence of an adequate amount of tachyzoites. Ultrathin sections (80 nm) contrasted with 4% uranyl acetate and Reynold’s lead citrate were observed with a Philips EM208S transmission electron microscope, operating at an accelerating voltage of 80 kV.

### Statistical Analysis

All data are reported as mean values ± SD from replicate experiments. Data were evaluated by Student’s *t*-test or one-way ANOVA (Tukey’s or Dunnett’s multiple comparison *post hoc* tests) with GrapPad Prism 5.0 software. Values of *P* < 0.05 were considered as significant.

## Results

### KP Inhibits *T. gondii* Infection in Vero Cells

It was preliminarily verified by the MTT assay the lack of toxicity of KP on Vero cells. Mean absorbance values were similar with medium alone (1.066 ± 0.255) and in presence of KP at 50, 100, and 200 μg/mL (1.060 ± 0.267, 1.049 ± 0.266, and 0.901 ± 0.171, viable cells approximately 99.4, 98.4, and 84.5%, respectively).

**FIGURE 1:**
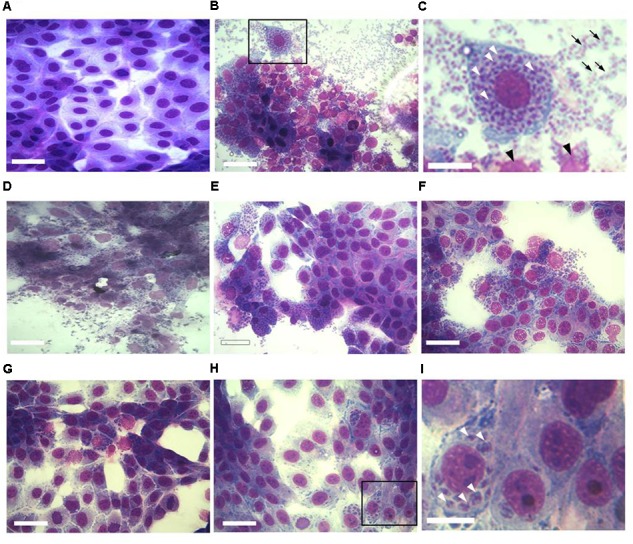
Effect of KP treatment on *Toxoplasma gondii* proliferation in Vero cells. Light microscopy images (40×) of Vero cells cultured in chamber slides and stained with modified Giemsa are shown. **(A)** Uninfected cells, bar: 200 μm; **(B)** cells infected with *T. gondii* in absence of KP, bar: 200 μm; **(C)** higher magnification of the inset in **(B)**, showing the high number of intracellular (white arrowheads) and extracellular (black arrows) parasites and debris of lysed cells (black arrowheads), bar: 100 μm; **(D)** cells infected with *T. gondii* in presence of the scrambled peptide SP, bar: 200 μm; **(E–H)** cells infected with *T. gondii* in presence of KP at increasing concentrations (25, 50, 100, and 200 μg/mL), bars: 200 μm; **(I)** higher magnification of the inset in **(H)**, showing a low number of intracellular parasites (white arrowheads) and the absence of extracellular tachyzoites, bar: 100 μm.

**FIGURE 2:**
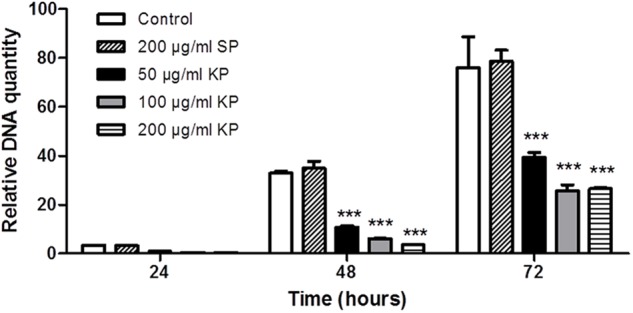
Effect of KP treatment on the efficiency of *T. gondii* infection in Vero cells, as measured by quantitative real-time PCR. Vero cells were infected with *T. gondii* in absence (control), and in presence of KP (50, 100, and 200 μg/mL), or the scrambled peptide SP (200 μg/mL). After 3 h (*T_0_*), the medium was replaced with fresh RPMI (2% FBS) without peptides. At 24, 48, or 72 h post-infection, the amount of *T. gondii* DNA in each sample was determined targeting a 94-bp internal fragment in a 529-bp repeat element of the parasite genome (Accession No.: AF146527). DNA amount is expressed as relative quantity in comparison to DNA obtained at *T_0_.* Data reported are the mean values ± SD from three replicate wells (^∗∗∗^*P* < 0.001 compared to control, as determined by one-way ANOVA with Tukey *post hoc* test).

The effect of increasing concentrations of KP on *T. gondii* proliferation in Vero cells was investigated by light microscopy and real-time PCR. Infection index and intracellular proliferation were significantly reduced by KP (**Table 1**). Infection index showed a decrease ranging from 33 to 87% at KP concentrations of 50 and 200 μg/mL, respectively. Intracellular parasite proliferation was similarly reduced, with a decrease of 54 and 95% at 50 and 200 μg/mL of KP, respectively. The scrambled peptide SP had no significant effect on either infection index or parasite proliferation. Representative images of the analyzed samples are shown in **Figure 1**. Real-time PCR showed a significant reduction of parasite DNA in cells infected in presence of KP for 3 h. As shown in **Figure 2**, intracellular parasite proliferation at 48 and 72 h post-infection was lower in cells infected in presence of KP than in untreated cells or cells infected in presence of SP.

**Table 1:** Effect of KP treatment on infection index and intracellular proliferation of *T. gondii* in Vero cells.

Treatment	Infection index^a^		Intracellular proliferation^b^	
	Mean ±*SD*	% Inhibition *vs.* control	Mean ±*SD*	% Inhibition *vs.* control
Control^c^	175 ± 3	-	3368 ± 1123	-
KP 25 μg/mL	118 ± 22***	33	1557 ± 225**	54
KP 50 μg/mL	106 ± 16***	39	858 ± 130***	75
KP 100 μg mL	42 ± 5***	76	253 ± 100***	92
KP 200 μg/mL	22 ± 17***	87	181 ± 45***	95
SP 200 μg/mL	158 ± 7	9	2315 ± 779	31


*^*a*^Infection index: number of infected cells per 200 examined cells; ^*b*^intracellular proliferation: number of parasites per 200 examined cells; ^*c*^medium alone. SP: scrambled synthetic peptide containing the same amino acids of KP in a different order. ^∗∗^*P* < 0.01, ^∗∗∗^*P* < 0.001, as determined by one-way ANOVA with Dunnett *post hoc* test *vs.* control.*

### KP Triggers Phosphatidylserine Externalization in *T. gondii* Without Membrane Damage

**FIGURE 3:**
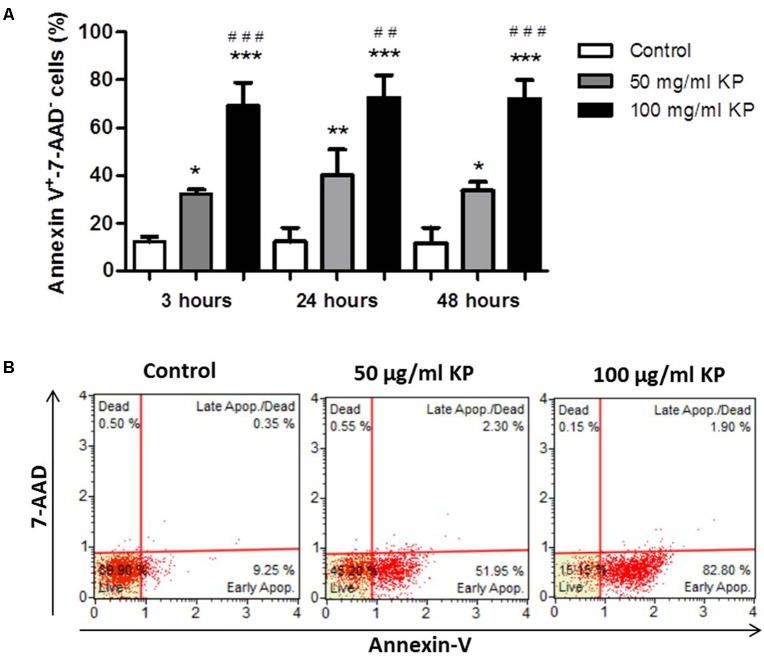
Effect of KP treatment on phosphatidylserine exposure in extracellular *T. gondii* tachyzoites. **(A)** Percentage values of Annexin-V^+^/7-AAD^-^ parasites after the indicated periods of incubation with KP at 50 and 100 μg/mL and medium alone (control). Data are expressed as mean values ± SD from at least three independent experiments. ^∗^*P* < 0.05, ^∗∗^*P* < 0.01, ^∗∗∗^*P* < 0.001, *vs.* control; ^##^*P* < 0.01, ^###^*P* < 0.001, *vs.* treatment with KP at 50 μg/mL; assessed by one-way ANOVA with Tukey *post hoc* test. **(B)** Representative two dimensional dot plots obtained after incubation of tachyzoites for 48 h with KP or medium alone (control).

After treatment with KP for 3 h, a significant, dose-dependent increase in the percentage of Annexin-V^+^/7-AAD^-^ cells, indicating the induction of PS externalization without loss of membrane integrity, was observed. Prolonged KP exposure (24 and 48 h) did not increase the percentages of Annexin-V^+^/7-AAD^-^ cells (**Figure 3A**). At all investigated times, the percentages of 7-AAD^+^ cells were low, less than 2.5% in each tested condition after 48 h of incubation (**Figure 3B**), thus excluding a prominent induction of cell membrane destruction.

### KP Exposure Leads to *T. gondii* DNA Fragmentation

**FIGURE 4:**
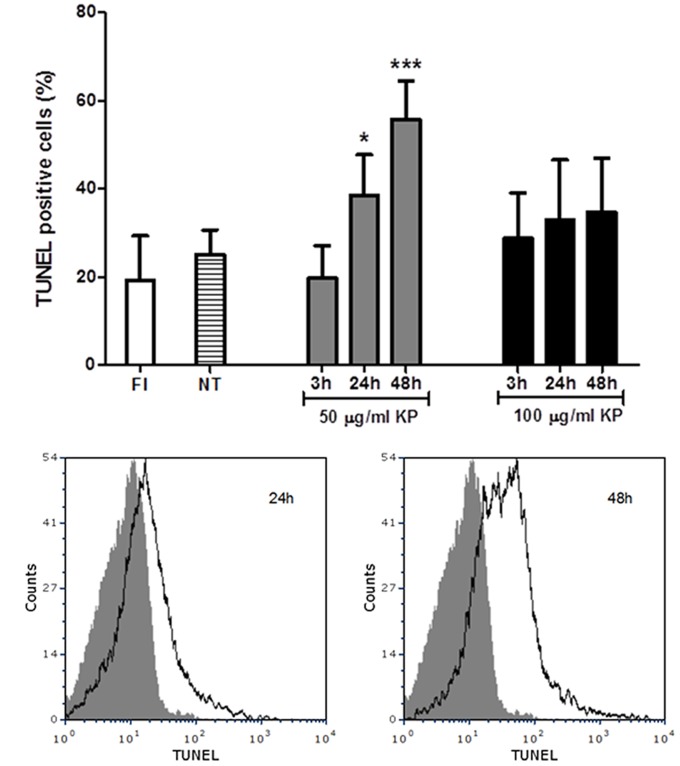
Effect of KP treatment on DNA fragmentation in extracellular *T. gondii* tachyzoites. DNA strand breaks were detected with an *in situ* TUNEL assay and analyzed by flow cytometry. **(A)** Percentages of TUNEL positive cells detected in freshly isolated tachyzoites (FI, white bar) and in tachyzoites treated with KP at 50 (gray bars) or 100 μg/mL (black bars) for the indicated periods of time. Percentages of TUNEL positive cells in tachyzoites incubated with medium alone at each time point (non-treated controls) are reported as an average (NT, lined bar). Data are expressed as mean ± SD from two independent experiments, and compared with Student’s *t*-test to NT value (^∗^*P* < 0.05, ^∗∗∗^*P* < 0.001). **(B)** Representative TUNEL fluorescence histograms of *T. gondii* obtained after treatment with KP at 50 μg/mL. Solid histogram: freshly isolated tachyzoites; open histogram: tachyzoites after 24 or 48 h of treatment.

A flow cytometry analysis of *T. gondii* tachyzoites stained for fragmented DNA using a TUNEL assay was performed. While an average of 19% freshly isolated parasites were positive at TUNEL assay, TUNEL positive cells were 25%, on average, after incubation in medium without KP for 3, 24, and 48 h (**Figure 4A**). Cells with fragmented DNA were significantly increased after treatment with 50 μg/mL KP for 24 h (39%) and 48 h (56%). In tachyzoites treated with 100 μg/mL KP, percentages of TUNEL positive cells were not significantly different from control, and the reactivity for DNA breaks did not significantly increase overtime. Representative TUNEL fluorescence histograms of *T. gondii* obtained after treatment with KP at 50 μg/mL are shown in **Figure 4B**.

### KP Induces Dissipation of Mitochondrial Membrane Potential in *T. gondii*

**FIGURE 5:**
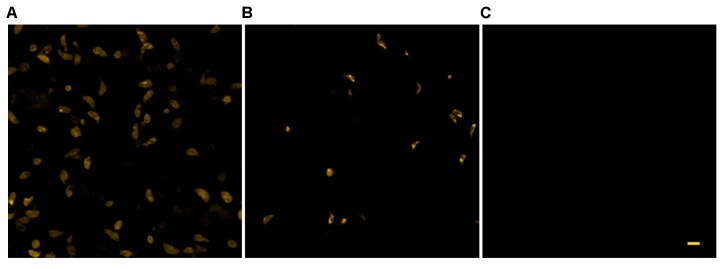
Effect of KP treatment on TMRM loading in extracellular *T. gondii* tachyzoites. Representative confocal images of TMRM labeled tachyzoites incubated for 18 h in the absence **(A)** or presence of 50 **(B)** and 100 **(C)** μg/mL of KP. Bar: 5 μm.

**FIGURE 6:**
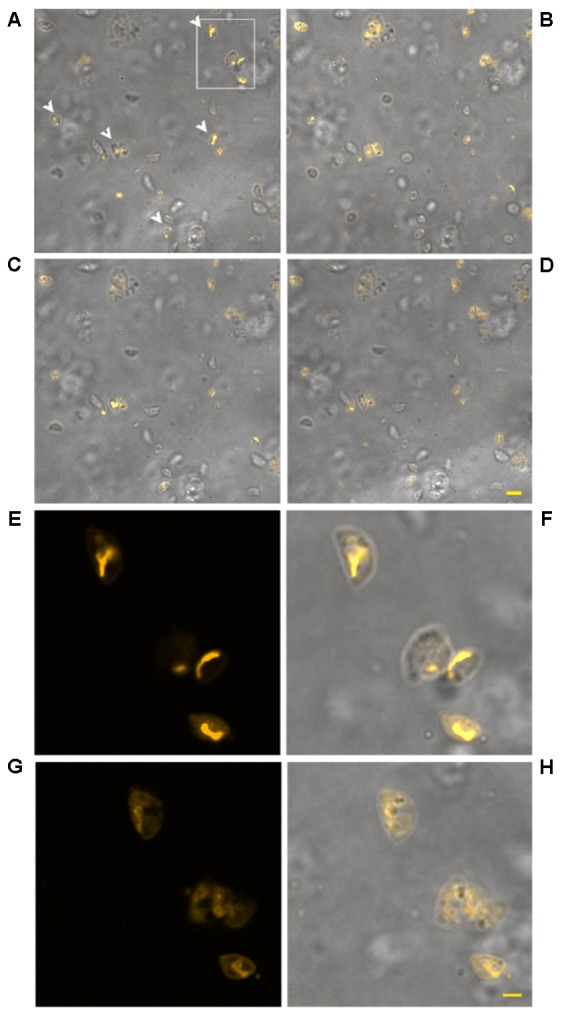
Effect of treatment with KP at 50 μg/mL on TMRM loading in extracellular *T. gondii* tachyzoites in a time-lapse assay. Confocal images of viable parasites labeled with TMRM before **(A)** and after incubation with the peptide for 10 min **(B)**, 1 h **(C)**, and 2 h **(D)**. The same field is shown. Signal of TMRM and transmitted light images are merged to highlight both TMRM distribution and tachyzoites morphology. Arrowheads in **(A)** indicate tachyzoites with stained mitochondria. Bar: 5 μm. The inset in **(A)** is presented at higher magnification in **(E)** (TMRM fluorescence) showing the structure of *T. gondii* mitochondrion, and the position of the mitochondrion into the cell (**F**, merged image). The same inset after 3 h of incubation with the peptide is presented, showing a low residual TMRM fluorescence localized outside the mitochondrion **(G)** and prominent alterations of tachyzoites morphology (**H**, merged image). Bar: 2 μm.

**FIGURE 7:**
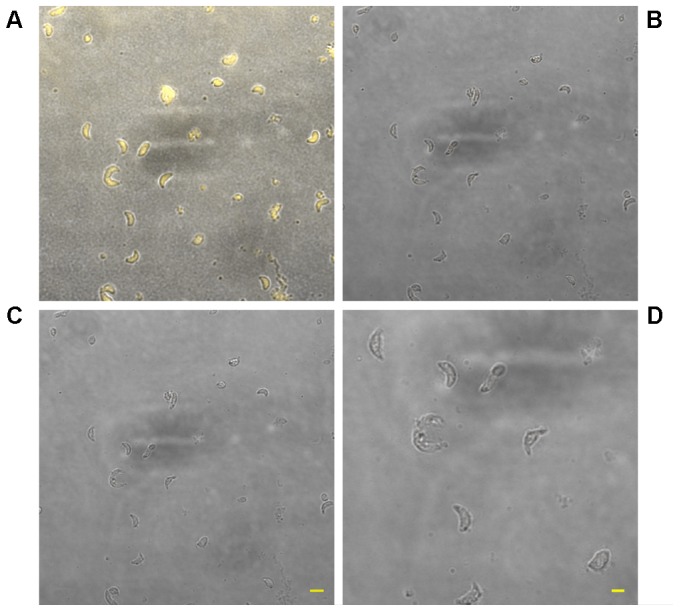
Effect of treatment with KP at 100 μg/mL on TMRM loading in extracellular *T. gondii* tachyzoites in a time-lapse assay. Confocal images of viable parasites labeled with TMRM before **(A)** and after incubation with the peptide for 15 min **(B)** and 1 h **(C)**. Bar: 5 μm. The same field is shown. Signal of TMRM and transmitted light images are merged. A complete loss of TMRM signal indicative of ΔΨm collapse was recorded after 15 min of treatment. Higher magnification of the tachyzoites in **(C)** shows details of the altered morphology and cell shrinkage induced by the peptide **(D)**. Bar: 2 μm.

The use of TMRM as a potentiometric probe was preliminarily validated by demonstrating its ability to label specifically *T. gondii* mitochondrion. A lack of fluorescence without alterations in cell morphology, indicative of complete loss of ΔΨm, was observed 4 min after the addition of FCCP. Changes in ΔΨm of tachyzoites exposed to KP were then evaluated by TMRM loading. After 18 h incubation in medium alone tachyzoites retained the ability to sequester TMRM in the mitochondrion (FI 100%), indicating an intact ΔΨm (**Figure 5A**). Instead, following 18 h of incubation with 50 μg/mL KP, TMRM loading was significantly reduced (FI 27.4%, *P* < 0.01 as assessed by Student’s *t*-test) (**Figure 5B**). Tachyzoites incubated with 100 μg/mL KP failed to sequester TMRM (no fluorescence recorded), indicating a complete loss of ΔΨm (**Figure 5C**). Freshly isolated tachyzoites loaded with TMRM were also monitored by CLSM in time-lapse experiments up to 3 h after exposure to KP. Images of the same field, acquired before and after KP addition (50 μg/mL), revealed a progressive decrease in probe loading, indicative of a gradual loss of ΔΨm (**Figures 6A–D**). Before KP treatment, TMRM accumulated in the single mitochondrion, highlighting its tubular shape (**Figures 6E,F**). After 3 h of exposure to KP, TMRM was released from the mitochondrion and diffused into the cytoplasm (**Figures 6G,H**). A complete loss of TMRM fluorescence, indicative of ΔΨm collapse, was recorded in tachyzoites after 15 min of treatment with KP at 100 μg/mL (**Figures 7A–C**). Monitoring for 60 min showed that ΔΨm was not restored. By observation in transmitted light, parasite morphology appeared altered with irregular borders and cell shrinkage (**Figure 7D**). Time-lapse observation of tachyzoites loaded with TMRM and maintained in medium alone revealed that mitochondria retained an intact ΔΨm, without significant variations over time (data not shown).

### KP Treatment Causes Ultrastructural Changes in *T. gondii* Tachyzoites

**FIGURE 8:**
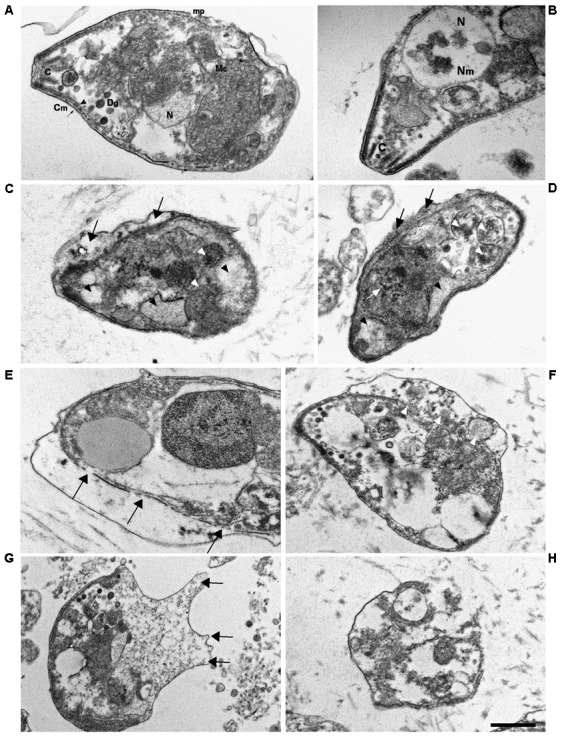
Ultrastructural alterations in *T. gondii* tachyzoites after KP treatment. TEM images were obtained after 3 h incubation in absence (control) or presence of KP. **(A)** Typical morphology of extracellular parasites incubated in absence of KP (control). C, conoid; mp, micropore; Mc, mitochondrion; N, nucleus; Dg, dense granule; Cm, cell membrane (plasmalemma, arrow; inner cell membrane complex, arrowhead). **(B)** Higher magnification of the apical pole of a control parasite. C, conoid; Nm, nuclear membrane; N, nucleus. **(C,D)** Morphological changes after 3 h of incubation with KP at 50 μg/mL. Rupture of nuclear membrane and chromatin condensation (white arrow), disorganization of the mitochondrion (white arrowheads), formation of vacuoles, either phase lucent or containing small granules (black arrowheads), and detachment of plasmalemma (black arrows). **(E–H)** Gross alterations of cellular structures after 3 h of incubation with KP at 100 μg/mL. **(E)** Inner membrane complex damages (arrows). **(F)** Cellular material shed in the space between detached membranes (white arrowheads).**(G)** Protrusions of plasmalemma containing amorphous material (arrows). **(H)** Almost completely lysed tachyzoite. Bar: 0.5 μm.

Transmission electron microscopy (TEM) images of tachyzoites incubated in medium alone for 3 h showed the normal crescent-shaped morphology, with intact nuclear and cell membranes; the characteristic double-layered inner cell membrane complex was visible (**Figures 8A,B**). Conoids, rhoptries, and micronemes were distributed orderly toward the apical pole. Dense granules and intact mitochondria were also observed. By contrast, most tachyzoites treated for 3 h with 50 μg/mL KP presented an altered morphology, with cytoplasmic shrinkage, disruption of nuclear membrane, chromatin condensation, and loss of mitochondrion structure (**Figures 8C,D**). Reduction in the number of dense granules, increase in cytoplasmic vacuolation, blur of the conoid, and detachment of the plasmalemma were also observed. After treatment with 100 μg/mL KP, the majority of tachyzoites were completely destroyed. Images from cells partially preserved revealed an apparent initial disruption of the inner membrane complex, with shedding of cellular material in the space underlying the detached plasmalemma before complete rupture of membranes (**Figures 8E–H**).

## Discussion

In the present study, the synthetic decapeptide KP demonstrated its activity against *T. gondii* tachyzoites. Importantly, effective KP concentrations proved to be non-toxic to Vero cells, in agreement with previous demonstrations of lack of detectable toxicity to other cell lines, human peripheral blood mononuclear cells, and erythrocytes (Magliani et al., 2011). Previous studies demonstrated an *in vitro* time- and concentration-dependent cytocidal effect of KP against *A. castellanii* trophozoites and *Leishmania* spp. promastigotes (Fiori et al., 2006; Savoia et al., 2006). Potential targets of KP in these pathogens have been identified as superficially-expressed β-1,3-glucan receptors. These glucans have not been described in *T. gondii* tachyzoites, although they are present on the membrane of oocysts (Bushkin et al., 2012). Killing of *L. infantum* promastigotes by KP involved alterations consistent with an autophagic cell degeneration, possibly induced by KP interaction with the β-glucan receptors (Savoia et al., 2006).

Following treatment with different drugs and other stimuli, *T. gondii* showed to undergo phenotypic changes similar to those observed during apoptotic (Peng et al., 2003; Ni Nyoman and Lüder, 2013; Zeng et al., 2013; Li et al., 2015) or autophagic (Lavine and Arrizabalaga, 2012; Ietta et al., 2017) cell death in metazoans. Diverse approaches were used in the present study to assess if KP could trigger specific cell death pathways in *T. gondii*. After KP treatment, flow cytometry analysis using Annexin-V and 7-AAD as probes showed a rapid and dose-dependent PS externalization without prominent loss of plasma membrane integrity. PS externalization without significant rupture of plasma membrane was previously observed following treatment of *T. gondii* with chemotherapeutic agents, using Annexin-V and either 7-AAD (Ni Nyoman and Lüder, 2013) or propidium iodide (Mikaeiloo et al., 2016) as probes for membrane permeabilization. The lack of increase in Annexin-V positive tachyzoites over time was also observed (Ni Nyoman and Lüder, 2013). PS externalization is an early event in apoptotic death of mammalian cells (Kroemer et al., 2009). Although not universally accepted, positivity to Annexin-V has also been proposed as a marker to define apoptosis-like events in protozoan parasites (Jiménez-Ruiz et al., 2010; Proto et al., 2013). However, increased Annexin-V binding might not necessarily be indicative of apoptotic cell death in *T. gondii* and *Leishmania*, as PS exposure in these protozoa was also associated with a conserved adaptation mechanism for apoptotic mimicry and evasion of immune response of activated macrophages (Seabra et al., 2004; Wanderley et al., 2006; dos Santos et al., 2011). To confirm an apoptotic phenotype in KP-treated tachyzoites, other biochemical and morphological markers were therefore evaluated.

Dissipation of ΔΨm together with activation of caspase-like proteases and high expression levels of putative pro-apoptotic regulators was associated to apoptosis-like cell death induced by miltefosine treatment in *T. gondii* (Ni Nyoman and Lüder, 2013). After treatment of tachyzoites with KP, we determined changes in ΔΨm using TMRM, already exploited in *L. infantum* (Alzate et al., 2006) and *Trypanosoma brucei* (Figarella et al., 2005) as a potentiometric fluorescent probe to show dissipation of ΔΨ_m_ in response to different stresses. We validated TMRM use in *T. gondii* showing a specific mitochondrial labeling and the ability of this probe to detect changes in ΔΨ_m_. Tachyzoites showed an impaired ability to sequester TMRM after overnight incubation with KP at 50 μg/mL, and a complete loss of mitochondrial labeling after treatment with double peptide concentration. Time-lapse experiments confirmed a partial loss of ΔΨm starting 10 min after the addition of 50 μg/mL KP and a complete collapse of ΔΨm in 15 min after exposure to 100 μg/mL KP.

Another key event of apoptosis is endonucleolysis, which results in the cleavage of nuclear DNA into oligonucleosome-sized fragments (Elmore, 2007). Fragmentation of *T. gondii* genomic DNA after 20 h of exposure to nitric oxide was first demonstrated by electrophoresis (Peng et al., 2003). A TUNEL assay was also used to detect DNA strand breaks in *T. gondii* after treatment with pro-apoptotic stimuli, using CLSM to confirm that position and size of TUNEL-reactive structures were compatible with staining of parasite nuclei (Ni Nyoman and Lüder, 2013). DNA strand breaks were detected starting from 24 h of treatment. After treatment with KP at 50 μg/mL, reactivity for DNA strand breaks appeared to be time-dependent, increasing from basal levels (nearly 20% positive cells) detected after 3 h of incubation, to 39 and 56% after 24 and 48 h, respectively, confirming that DNA fragmentation is a late event. Interestingly, in presence of KP at 100 μg/mL no significant increase in TUNEL positive cells was detected at any time point. A possible explanation is that, in these conditions, nuclei had undergone too extensive or complete fragmentation, which could not be detected by TUNEL assay.

Morphological changes, previously associated with apoptosis, were observed by TEM in KP-treated tachyzoites, including cytoplasmic shrinkage and vacuolation, blur of conoid, loss of mitochondrion structure, disruption of nuclear membrane, and chromatin condensation. In particular, the observation of condensed chromatin, which in mammalian apoptotic cells has been demonstrated to morphologically consists of endogenously digested chromatin fragments (Wyllie et al., 1984), is in agreement with the results obtained in TUNEL assays. Detachment of plasmalemma was also detected. At higher KP concentration, TEM images of cells not completely destroyed suggested an initial disruption of the inner membrane complex, with shedding of amorphous cellular material in the space underlying the detached plasmalemma before complete cell rupture. This phenomenon could partly explain the exclusion from the cell of 7-AAD.

Although molecular studies should be needed to entirely exclude that autophagic phenomena could be induced by peptide treatment, the obtained data strongly suggest that KP may induce an apoptosis-like cell death in *T. gondii* tachyzoites. Recent researches demonstrated that molecular pathways that characterize *T. gondii* apoptosis-like cell death only partially overlap with those of higher eukaryotes (Ni Nyoman and Lüder, 2013; Li et al., 2015). Antimicrobial compounds triggering programmed cell death in *T. gondii* may thus be useful drug candidates. In conclusion, KP could be considered a promising compound for the development of novel and safe anti-*Toxoplasma* agents. Further studies are needed to characterize the molecular target of KP and its activity against different parasite forms (e.g., bradyzoites). The therapeutic activity of KP, either alone or in combination with conventional drug, in animal models of toxoplasmosis should also be evaluated.

## Author Contributions

LG, LP, and LK conceived and designed the experiments. LG, CS, CM, AV, SB, TD, CF, TC, CB, WM, and SC performed the experiments and analyzed the data. LG, LP, SC, and LK wrote the paper. All authors reviewed and approved the final version of the manuscript.

## Conflict of Interest Statement

The authors declare that the research was conducted in the absence of any commercial or financial relationships that could be construed as a potential conflict of interest.
